# Protocol of a mixed method research design to study medical respite programs for persons experiencing homelessness

**DOI:** 10.1371/journal.pone.0295543

**Published:** 2024-01-11

**Authors:** Timothy P. Daaleman, Joanne Johnson, Lillian Gelberg, Robert Agans, Donna J. Biederman

**Affiliations:** 1 Department of Family Medicine, University of North Carolina at Chapel Hill, Chapel Hill, NC, United States of America; 2 Department of Health Behavior, Gillings School of Global Public Health, University of North Carolina at Chapel Hill, Chapel Hill, NC, United States of America; 3 Department of Family Medicine, UCLA David Geffen School of Medicine, Los Angeles, CA, United States of America; 4 Department of Health Policy & Management, UCLA Fielding School of Public Health, Los Angeles, CA, United States of America; 5 Department of Biostatistics, Gillings School of Global Public Health, University of North Carolina at Chapel Hill, Chapel Hill, NC, United States of America; 6 School of Nursing, Duke University, Durham, NC, United States of America; Dayeh University, TAIWAN

## Abstract

Medical Respite Programs (MRPs) characterize a care model that has been developed to address the health care and social needs of persons experiencing homelessness by providing post-acute hospital care in a safe environment. Although this model has been shown to reduce hospitalizations, improve health outcomes and increase access to health services, prior studies of MRP programs and outcomes have been limited to individual sites and may not generalize to the population of individuals receiving MRP care. This study protocol describes a mixed method design to collect organizational, provider, and patient-level data from a sample of MRPs.

## Introduction

Medical respite programs (MRPs) characterize a care model developed to address health care and social needs of persons experiencing homelessness (PEH) ([1–5]). MRPs provide acute and post-acute care for PEH who do not require hospitalization, but are too medically vulnerable to recover from a physical illness or injury in emergency shelters or on the streets ([1, 3, 6]). A randomized trial, for example, reported that a medical transition program paired with permanent supportive housing reduced hospitalizations by 29%, and ED visits by 24% for PEH ([7]). However MRPs vary in the services provided, their organizational models, and the patient populations that they serve; these data may not generalize to the MRP experience for other PEH receiving care ([2]). A 2013 systematic review concluded that MRPs can promote health outcomes and facilitate access to health services for PEH, but demonstrating the value of this care model and identifying best practices are limited by the quality of existing research ([4]).

The US Interagency Council on Homelessness recognized the potential of MRPs to improve health outcomes and reduce costs by highlighting this model in its 2015 Federal Strategic Plan to Prevent and End Homelessness ([1]), but research that examines health services in MRPs is limited to evaluations of individual programs and descriptive studies. For example, the Health Resources and Services Administration (HRSA) conducted a descriptive evaluation that reported variation in MRP settings (e.g., shelters, apartments, stand-alone facilities) and highlighted the complex care needs of the PEH population, but it did not examine MRP-level factors that impacted best practices and PEH outcomes ([8]). Another VA-based study focused on the primary care experience of PEH ([9]), however these findings do not generalize across MRPs and the evidence base for these care settings remains underdeveloped.

The provision of housing is a primary strategy to mitigate homelessness in the US with a Housing First (HF) approach that prioritizes securing permanent housing to PEH and provides a platform to improve health, quality of life, and access to health care services ([10–13]). Although MRPs align with this overall strategy by providing individuals with housing and engagement with medical, mental health and other services ([12, 14]), the paucity of health services data and absence of robust methods restricts the growth of this care model ([4, 5]). Emerging and established programs for PEH will need to identify and disseminate best practices in an expanding value-based care environment ([5, 15, 16]). In response, we describe a mixed method protocol to: (1) test the feasibility of collecting data from MRPs including MRP staff and PEH recipients of MRP care, and to; (2) describe MRP organizational-level elements, provider and patient characteristics, processes of care, and outcomes.

## Methods

### Conceptual framework

The study is informed by the Behavioral Model for Vulnerable Populations (BMVP), which is the primary conceptual model in the field of health care for PEH ([17]). The model includes individual level factors and contextual factors that highlight structural and enabling resources and has several domains. MRP-relevant variables at the individual level might comprise the following: the *Predisposing* domain includes demographic characteristics and housing/homelessness history, while the *Enabling* domain includes self-efficacy and level of social support ([17]). The *Need* domain includes current functional, physical and mental health status and substance use, while *Behaviors* include engagement with health care services and chronic disease self-management ([17]).

At the contextual level (e.g., organizational, larger social environment) MRP-relevant variables comprise the following: the *Predisposing* domain includes geographic location while the *Enabling* domain includes the structure and organization of health care services, such as the provision of mental health and substance use treatment services, processes of care, and the characteristics of health care personnel ([17]). The *Need* domain includes the area availability of housing resources. MRP-related *Outcomes* include treatment completion and housing disposition at discharge from the MRP.

### Identification of study site sample and recruitment

The sampling frame was a convenience sample of three sites that were nominated by the National Health Care for the Homeless Council Respite Care Providers’ Network (RCPN) ([18]), had organizational stability, and agreed to participate. To account for variability in MRP settings, we sampled from different MRP practice types (e.g., freestanding medical respite unit; shelter-based model; and home and apartment-based. The administrator at candidate MRP sites was contacted via email, followed within 1 week by a video/telephone call to explain the study and assess interest in participation.

MRPs that agreed to participate had a video/telephone call to discuss the study methods, Internal Review Board (IRB) concerns, and to identify a facility liaison for the study. The liaison was responsible for disseminating the recruitment brochure to patients and staff, and coordinating communication between the research team and survey participants as needed. Facility liaisons did not ask for consent, answer questions about the study, or otherwise act as a representative of the study, but were allowed to participate in the study as a site administrator, provider, or staff member as self-designated by their responsibilities at the MRP.

### Study instruments

The BMVP informed item and measure selection for the data collection instruments. We developed and maintained self-administered Qualtrics web-based surveys for MRP providers (e.g., licensed medical providers) and staff. We used computer assisted telephone surveys for MRP administrators and patients due to potentially sensitive items (e.g., MRP budget, patient reported substance use disorder). In addition, we developed a monthly program summary data census report of all patients, and a patient chart review document to abstract data from the MRP medical record of site patients who participated in the study. Tables 1–3 represents the data tables.

**Table 1:** Medical respite program data elements: Individual characteristics.

Data element	Variable	Measure	Source
*Individual Characteristics*			
*Predisposing*	Age	Year of birth	Patient interview & MRP medical record review
	Language	Native English/English 2^nd^ language/no English	
	Sex assigned at birth	Male/female	
	Gender	Male/female/non-binary/transgender male/transgender female	
	Race	American Indian/Alaskan Native/Asian/Black/African-American/Native Hawaiian or other Pacific Islander/White	
	Ethnicity	Hispanic/non-Hispanic	
	Marital status	Never married/married/widowed/separated/ divorced/ Lifetime partner	
	Level of education	Less than HS/some HS/HS grad/some college/college grad	
	Cigarette use	Never/former smoker/current smoker	
	Housing prior to MRP	Shelters/transitional/outdoors/friends or family/own home	
	Homelessness experience	In the past three years, how many times have you experienced homelessness?	
	Communication barriers	Dementia/confused sedation/language barrier	Chart review
*Enabling*	Self-efficacy	4-items, Likert scale (1 = True, 5 = False): I think I get sick more than others/I am as healthy as anybody I know/I expect my health to get worse/I believe that my health is excellent or very good [24]	Patient interview
	Social Support	Adapted Duke-UNC, 8 items, Likert scale (1 = as much as I would like, 6 = not enough): I have people who care what happens to me/I get love and affection/I get chances to talk to someone about my problems/I can talk to someone I trust about personal and family problems/I get useful advice/I get help when I am sick [25]	
	General health status	Self-rated health, Likert scale (1 = excellent, 5 = poor) [26]	Patient interview & MRP medical record review
*Need*	Functional status	10-items, Likert scale (1 = Limited, 3 = not limited): vigorous activities/moderate activities/lifting or carrying/climbing several flights of stairs/climbing one flight of stairs/bending or kneeling/walking more than a mile/walking several blocks/walking one block/bathing or dressing [27]	
	Mental health status	MHI-5 items, Likert scale (1 = all the time, 6 = never): How much of the time in the last month have you felt happy/calm and peaceful/nervous/sad/depressed? [28]	
	Trauma exposure	PC-PTSD-5 items (yes/no): In the last month have you had nightmares/tried not to think about events or avoided situations that remind you of them/been constantly on guard/felt numb or detached/felt guilty or blamed yourself or others for problems [29]	
	Mental health and substance use disorders	Mental health and substance use disorder diagnoses (from MRP medical record)	
	Physical health conditions	Medical diagnoses (from MRP medical record)	

**Table 2:** Medical respite program data elements: Contextual characteristics.

Data element	Variable	Measure	Source
*Contextual Characteristics*			
*Predisposing*	Characteristics of the external environment	Geographic designation (urban, suburban, etc.)	Administrator Interview
		Distance to nearest: hospital, urgent care, emergency dept.	
*Enabling*	Organization of MRP health care services	Frequency of MRP visits	Provider survey
		Reimbursement for MRP visits	
		Medical/health records (EHR, etc.)	
		Liability concern when treating MRP patients	
		Confidence in MRP staff	
		Medication management practices	Provider and staff surveys
		Acute problem management practices	
		Transfer practices	
		Working relationships (15 items)	
		Provider/staff satisfaction	
	Provider/Staff Characteristics:		
	Age	Year of birth	
	Gender	Male/female/non-binary/transgender male/transgender female	
	Ethnicity	Hispanic/non-Hispanic	
	Race	American Indian/Alaskan Native/Asian/ Black/ African-American/Native Hawaiian or other Pacific Islander/White	
	Certification	Providers: RN/LPN/MD/PA/SW/Pharmacy	
		Facility Staff: administrator/CNA/nurse aide or assistant/peer support specialist	
	Experience	Hours worked per week; Time working with people experiencing homelessness	
	Characteristics of MRP organization	Program policies & best practices	Administrator Interview
		Facility ownership	
		MRP model (stand alone, shelter, etc.)	
		Years in operation	
		Beds (occupancy, specified treatment)	
		Patient age, race & ethnicity	
		Patient payor source	
		Staffing (RN, LPN, paid/unpaid)	
		Contracted services	
		Medical/health records	
		Financial model and budget	
		Partnerships with Community Service Providers	
		Continuum of Care (CoC) Housing Inventory Count including; emergency shelter, transitional housing, safe-haven, and permanent housing [30]	HUD website

**Table 3:** Medical respite program data elements: Behaviors and outcomes.

Data element	Variable	Measure	Source
*Behaviors*			
*Individual*	MRP healthcare utilization	Frequency: daily/weekly/sporadically/not at all	Patient interview & medical record review
		Type including; medical provider, nursing, social work, occupational therapy, substance use counselor, mental health counselor	
	Disease self-management	Adapted PETS, 2-items, Likert scale (1 = very much, 5 = not at all): Difficulty of keeping track of health conditions/Difficulty of monitoring health behaviors [31]	Patient interview
*Outcomes*			
*Individual*	Housing status at discharge	Hospital/emergency room??	MRP Monthly summary reporting form
		Shelters/transitional/outdoors/friends or family/own home	
*Facility level*	Premature discharge	Number of clients discharged prematurely/AMA	Monthly summary reporting form

### Data collection

After agreeing to participate, we mailed the facility liaison a packet of study brochures and copies of consent forms, and study incentives to distribute to patients. A research assistant (RA) was designated as the primary contact for the study site. After verbal, informed consent by the facility liaison, the RA collected facility-level measures via phone interviews with the site administrator (i.e., individual self-identified as responsible for MRP operations) and entered the data into a web-based survey. We sent the participating facility liaison the survey questions and consent form one week before the phone interview.

The facility liaison provided the research team with the names and email addresses of providers and staff at their facility who agreed to participate after reviewing consent forms and study brochures. The providers and staff (approximately 3–5 per site) received an email with a link to informed consent and the web-based survey (i.e., provider and staff survey) from the RA and confirmed via email when completed. The facility liaison also identified a convenience sample of 10 MRP patients as candidates to be interviewed. Responses were coded with identification numbers, not names, and the list linking numbers and names were kept in a separate file. No information provided was given to the treating provider or MRP staff. Since not all patients had access to individual phones, the interviews were conducted on a secure phone line at the facility. After verbal informed consent, responses to the computer facilitated telephone interview were entered into the database.

Patient consent also included a HIPAA authorization form granting permission for study investigators to access medical records for research purposes. After completion of patient interviews, the research team worked with the facility liaison in collecting, copying, and transferring medical records, from the time of admission to the time of data collection, to a HIPAA compliant fax line, which was entered into a web based Qualtrics database. At four and eight weeks after completion of patient surveys, the facility liaison was asked to complete an electronic monthly facility-level report that included total MRP census and patient discharge disposition.

### Ethics

The study was reviewed and approved by the University of North Carolina Chapel Hill Internal Review Board (IRB #21–0196). All participating sites received a $2000 honorarium and staff did not receive an incentive to participate. All patients who participated in the study received a $10 gift card.

### Data analyses

We will use descriptive statistics to determine the feasibility of the data collection methodology by comparing the participation and completion rates with a prior study of PEH, which is approximately 22% for patients ([19]). To our knowledge, there are no prior studies that have reported MRP study participation rates. We will use descriptive statistics to describe the study samples, Chi-square tests of independence and employ Fisher’s exact tests in cases of sparse data to examine the association between item response/nonresponse and facility characteristics, MRP provider/staff characteristics, and PEH characteristics.

We will examine the unadjusted associations between MRP organizational (based on the Administrator Survey), provider and staff characteristics (based on the Provide Survey), patient characteristics (based on the Patient Survey) and the outcomes of: (1) treatment completion and (2) enrollment into housing programs using chi-square test of independence and Fisher’s exact test. We will use Odd Ratios (ORs) to describe the magnitude of these associations. The primary goal of these future analyses will be to identify plausible relationships between selected facility and individual-level characteristics and outcomes of interest.

## Results and discussion

To our knowledge this protocol is the first description of a systematic, mixed method approach that collected data from medical respite programs (MRPs). Our sampling included administrators, providers, staff, and PEH receiving care in the MRP. Data elements were multi-level and included MRP organizational-level elements, provider and patient characteristics, processes of care, and outcomes. Site recruitment began in January 2022 and continued through August 2022. A total of 10 sites were contacted via email; 6 responded to the communication and 3 agreed to participate. Reasons for refusal included staff burden and COVID-19. Data collection at the 3 sites was completed in April 2023. We completed data collection for 10 PEH receiving care (total N = 30) and for 3–5 providers and staff (N = 11) at each respective MRP site.

We are unaware of prior reports of MRP participation rates. The previously described Health Resources and Services Administration (HRSA) evaluation did not report a rate and is not generalizable since MRP sites were required to participate as grant recipients ([8]). We will use our experience to refine our data collection methods in order to support a future national, representative sample of RCPN members. In addition, we are cleaning and analyzing the data to support assumptions regarding sample size and power estimates for the larger project by estimating the preliminary effects of patient characteristics and organizational level components on key outcomes. Such a study will be able to determine the components and characteristics of MRPs that contribute to identifying outcomes that are important to PEH, as well as outcomes such as treatment completion and enrollment into housing, can greatly promote the emerging evidence base in the field ([20]).

The strengths of the protocol include use of a robust conceptual model (i.e., BMVP) that informed the items and measures collected in the study. Our multilevel (e.g., individual, organizational, contextual) perspective and data collection from multiple data sources, including MRP provider and staff surveys, computer facilitated interviews of site administrators and patients, and medical record review, have been previously used in studies of long-term care [21].

Study limitations include the challenges to conducting research with PEH since they are often lack consistent contact information and communication resources when compared to the general population [22]. In addition, although we partnered with the National Institute for Medical Respite Care, we did not directly engage in persons experiencing homelessness in developing the protocol. Engaging this vulnerable population and their MRP care providers can require persistence and frequent contact [22].

## Conclusions

Although we determined that our protocol was feasible in collecting multiple sources of data from MRP sites, we identified challenges in communicating with MRP patients, staff and providers due to fallout from the COVID-19 pandemic, burnout in health care staff, higher workloads and additional psychosocial stressors [23]. The heaviest burden of care in MRPs falls on nursing assistants, medical assistants, social workers, and other direct care staff [23], who play key roles in providing care in MRP sites ([5]). We determined that staff shortages and burnout led to some MRP sites refusing participation or delaying data collection, which contributed to reduced response rates and missing data.

## Supporting information

S1 Checklist *PLOS ONE* clinical studies checklist. (DOCX)
